# Field Epidemiology and Laboratory Training Programs in sub-Saharan Africa from 2004 to 2010: need, the process, and prospects

**DOI:** 10.4314/pamj.v10i0.72235

**Published:** 2011-10-19

**Authors:** Peter Nsubuga, Kenneth Johnson, Christopher Tetteh, Joseph Oundo, Andrew Weathers, James Vaughan, Suzanne Elbon, Mufuta Tshimanga, Faustine Ndugulile, Chima Ohuabunwo, Michele Evering-Watley, Fausta Mosha, Obinna Oleribe, Patrick Nguku, Lora Davis, Nykiconia Preacely, Richard Luce, Simon Antara, Hiari Imara, Yassa Ndjakani, Timothy Doyle, Yescenia Espinosa, Ditu Kazambu, Dieula Delissaint, John Ngulefac, Kariuki Njenga

**Affiliations:** 1 Division of Public Health Systems and Workforce Development, Center for Global Health, US Centers for Disease Control and Prevention; 2 Previously Epidemiology Resident Advisor for the Kenya and South African Field Epidemiology and Laboratory Training Programs; 3 Laboratory Resident Advisor Kenya Field Epidemiology Training Program; 4 Zimbabwe Field Epidemiology Training Program (previously Epidemiology Resident Advisor, South Africa Field Epidemiology and Laboratory Training Program); 5 Member of Parliament, United Republic of Tanzania (previously Laboratory Resident Advisor, South Africa Field Epidemiology Training Program); 6 Resident Advisor Ghana Field Epidemiology and Laboratory Training Program; 7 Director, National Health Laboratory, Tanzania Ministry of Health and Social Welfare (previously Laboratory Resident Advisor Tanzania Field Epidemiology and Laboratory Training Program); 8 Previously Epidemiology Resident Advisor Tanzania Field Epidemiology and Laboratory Training Program; 9 Epidemiology Resident Advisor, Nigeria Field Epidemiology and Laboratory Training Program; 10 Veterinary Resident Advisor, Nigeria Field Epidemiology and Laboratory Training Program; 11 Previously Resident Advisor, Ethiopia Field Epidemiology and Laboratory Training Program; 12 Epidemiology Resident Advisor, Rwanda Field Epidemiology and Laboratory Training Program; 13 Resident Advisor, West African Field Epidemiology and Laboratory Training Program; 14 Resident Advisor, Mozambique Field Epidemiology and Laboratory Training Program; 15 Resident Advisor, Central African Field Epidemiology and Laboratory Training Program; 16 CDC Kenya and previously Laboratory Resident Advisor, Kenya Field Epidemiology and Laboratory Training Program

**Keywords:** Field epidemiology, laboratory management, multi-disease surveillance and response systems, public health workforce capacity building

## Abstract

As of 2010 sub-Saharan Africa had approximately 865 million inhabitants living with numerous public health challenges. Several public health initiatives [e.g., the United States (US) President's Emergency Plan for AIDS Relief and the US President's Malaria Initiative] have been very successful at reducing mortality from priority diseases. A competently trained public health workforce that can operate multi-disease surveillance and response systems is necessary to build upon and sustain these successes and to address other public health problems. Sub-Saharan Africa appears to have weathered the recent global economic downturn remarkably well and its increasing middle class may soon demand stronger public health systems to protect communities. The Epidemic Intelligence Service (EIS) program of the US Centers for Disease Control and Prevention (CDC) has been the backbone of public health surveillance and response in the US during its 60 years of existence. EIS has been adapted internationally to create the Field Epidemiology Training Program (FETP) in several countries. In the 1990s CDC and the Rockefeller Foundation collaborated with the Uganda and Zimbabwe ministries of health and local universities to create 2-year Public Health Schools Without Walls (PHSWOWs) which were based on the FETP model. In 2004 the FETP model was further adapted to create the Field Epidemiology and Laboratory Training Program (FELTP) in Kenya to conduct joint competency-based training for field epidemiologists and public health laboratory scientists providing a master's degree to participants upon completion. The FELTP model has been implemented in several additional countries in sub-Saharan Africa. By the end of 2010 these 10 FELTPs and two PHSWOWs covered 613 million of the 865 million people in sub-Saharan Africa and had enrolled 743 public health professionals. We describe the process that we used to develop 10 FELTPs covering 15 countries in sub-Saharan Africa from 2004 to 2010 as a strategy to develop a locally trained public health workforce that can operate multi-disease surveillance and response systems.

## Introduction

There were approximately 865 million people living in sub-Saharan Africa amidst multiple public health challenges and limited spending on public health in 2010 [1]. Achieving public health goals in sub-Saharan Africa remains an elusive target despite major donor funded initiatives, some of which have been widely successful. For example, the United States (US) President's Malaria Initiative (PMI) led to a marked reduction in malaria transmission in a few short years [2,3] and the US President's Emergency Plan for AIDS Relief (PEPFAR) markedly changed the fate of millions of HIV-infected people in the region and significantly reduced transmission of HIV from mothers to children [4]. Sub-Saharan Africa also suffers considerable preventable mortality from maternal deaths, deaths due to motor vehicle crashes, cardiovascular diseases, and cancers [5,6]. Effective public health surveillance and response systems allow countries to prioritize and address public health problems, but countries in the region have had challenges with developing robust systems and training the workforce to operate them [7]. Africa is currently experiencing an economic resurgence; its overall economic growth in 2010 was 4.75% (similar to that of India) – and several times that of developed countries and its projected growth in 2011 is 5.75%. Economists report that Africa appears to have escaped the worst of the global recession [8,9]. With an improving economic outlook there is likely a growing demand for effective public health surveillance and response systems that can protect communities from communicable and non-communicable disease conditions.

In order to strengthen public health surveillance and response systems in sub-Saharan Africa, there is a need to develop a public health workforce capacity with competencies in field epidemiology. In 1993, the Rockefeller Foundation collaborated with the ministries of health (MOH) of Ghana, Uganda, Zimbabwe, and Vietnam to develop Public Health Schools Without Walls (PHSWOWs) as novel public health training programs that were tailored to improve public health surveillance and response and health services management needs at national and sub-national levels [10,11]. Staff from the US Centers for Disease Control and Prevention (CDC) participated in the development of the Uganda and Zimbabwe PHSWOWs and both programs included a strong multi-disease surveillance and response training component which was based on CDC's Epidemic Intelligence Service (EIS) [12]. Both the Uganda and Zimbabwe PHSWOWs recruited EIS graduates as expatriate resident advisors to develop 2-year Masters of Public Health (MPH) granting public health leadership programs with field work that enabled the trainees to develop competencies in field epidemiology comparable to those that EIS provides. These competencies were also similar to those provided by Field Epidemiology Training Programs (FETPs) which were developed as an adaptation of EIS to international settings beginning in the 1980s [13–15]. Ghana developed a 1-year MPH granting PHSWOW program focusing on district health management with a limited field epidemiology component without an expatriate resident advisor. All three African PHSWOWs had no specific training for public health laboratory workers.

In 2003, the Ellison Medical Foundation, the CDC Foundation, and CDC, collaborated with the Kenya MOH to start an innovative joint 2-year public health leadership training program for field epidemiologists and public health laboratory epidemiologists (public health laboratory scientists who are trained in field epidemiology and public health laboratory management) with a mission to create partnerships to operate disease surveillance and response systems, investigate suspected outbreaks, and manage public health laboratory networks. The Kenya Field Epidemiology and Laboratory Training Program (FELTP) was designed as a regional program with participants from Kenya, Southern Sudan, Tanzania, and Ghana. Uganda only participated in the laboratory epidemiology track (as they have their own PHSWOW). Soon after inception, with its added value of a laboratory epidemiology track, the Kenya FELTP became the model of the new generation of field epidemiology programs in sub-Saharan Africa which are now all FELTPs [16].

In 2006, the public health threat posed by zoonoses particularly after human cases of avian influenza, and Severe Acute Respiratory Syndrome led to the recognition of the need to train veterinary epidemiologists in a specialty track in the FELTP. The intent was to create “one-health teams” who would be trained jointly to conduct surveillance and response to deal with the diseases occurring at the animal-human interface. To address this need, new and existing programs in sub-Saharan Africa started to train veterinarians along with physicians, other health scientists, and public health laboratory scientists to address zoonoses.

We describe the process that we used to develop 10 FELTPs covering 15 countries in sub-Saharan Africa over 6 years to develop a locally trained public health workforce that can operate multi-disease surveillance and response systems. By the end of 2010, these 10 FELTPs and the two PHSWOWs that were started in the 1990s covered an estimated 637 million of the 865 million people in sub-Saharan Africa and had enrolled 743 public health professionals.

## Methods

In 2003 we developed a standard process for implementing FELTPs as part of the creation of the first FELTP in Kenya. We have used this standard process for all the subsequent FELTPs in sub-Saharan Africa. Before starting the FELTP in each country, CDC received an invitation from the MOH which led to a cascade of activities, including: a) a pre-assessment visit, b) a formal assessment and program planning visit, c) identifying a funding source for the program plan, d) developing an MOH-led steering committee, e) developing and obtaining approval of the FELTP curriculum, f) recruiting key staff for the FELTP, g) implementing short courses in field epidemiology for key frontline staff and for screening of the initial cohort of FELTP participants, h) recruiting the first cohort of the FELTP, and i) implementing the 2-year FELTP. Each of these activities is described in detail below. For regional programs (FELTPs which involving more than one country) we adapted this process with more focus on the host country at startup of the regional FELTP.

The pre-assessment visits were conducted by a small team consisting of staff from CDC and existing FELTPs and PHSWOWs who visited the potential FELTP host country for about 1 week. The objectives of the visits were to gauge the interest of the MOH and its partners in starting an FELTP, to encourage the formation of an MOH-led Steering Committee, and to plan for the formal FELTP assessment and program planning visit. During the assessment and planning visits, conducted by a larger team usually 2-4 months after the pre-assessment, a representative sample of public health facilities at national and sub-national levels including public health laboratories were assessed using standard tools from the CDC FETP handbook to determine how they would participate in an FELTP [17]. Local universities were also assessed to determine their interest in the FELTP training model (which has a limited didactic period). If they were interested we determined the process to obtain a university-endorsed and approved FELTP curriculum that would be adapted from the standard core FETP curriculum developed by CDC [18]. Another team determined the cost of implementing an FELTP in the host country (and participating countries in a regional FELTP). At the end of each formal assessment we prepared an FELTP 5-year work plan including cost details and presented this to the first meeting of the FELTP Steering Committee. The Steering Committee, usually chaired by the highest ranking technical MOH official (e.g., the director of public health or chief medical officer), had representation from all the major stakeholders (e.g., CDC country office, World Health Organization representative, university senior leadership, public health laboratory service, veterinary service, and donors).

Implementation of the FELTP work plan began with the identification of resources for the program and selection of the university or universities to host the FELTP. In general most FELTPs began with a short course component to train frontline public health workers to address immediate field epidemiology, surveillance, outbreak investigation, and laboratory needs. The short courses were generally 2-weeks long and trained up to 30 participants per course. They were also used as screening courses for the first FELTP cohort, and were taught by a mix of international and regional FELTP and EIS graduates. Participants in the short courses had to develop and implement an applied learning project for 3 months at their worksites after the 2-week training as requirement for passing the course. These projects provided immediate benefit for the host country. Many countries conducted at least two short courses before starting the 2-year FELTP and these short courses continued along with the implementation of the FELTP.

The 2-year FELTP activities began with the recruitment of key staff, including at least one resident advisor who was usually a graduate of an FELTP or EIS or an experienced field epidemiologist, development of an FELTP office, finalization of the curriculum, and the advertisement and selection of the first cohort of the FELTP. The class size of the first cohort ranged from 10 to 15 with a mix of field epidemiology, public health laboratory, and veterinary candidates. The training strategy and competencies and expected outputs have been described elsewhere and are similar for all FELTPs in sub-Saharan Africa [16].

In 2005 we collaborated with the US Agency for International Development (USAID), the three PHSWOWs (i.e., Ghana, Uganda, and Zimbabwe), and the Kenya FELTP to develop and then support the African Field Epidemiology Network (AFENET) which is a networking and service alliance of African FELTPs [19–21]. AFENET, which is headquartered in Uganda, has become CDC's principal implementing partner for developing, staffing, and operating FELTPs in sub-Saharan Africa. CDC has worked with AFENET to plan and develop FELTPs, to participate in organizing short courses, and to implement regional initiatives for FELTPs (e.g., regional biennial scientific meetings and regional training fellowships).

CDC provided technical assistance from Atlanta in the operation of the FELTPs through program specific teams that included field epidemiologists (generally graduates of the CDC EIS Program), instructional designers, public health advisors, and health education specialists. The Atlanta teams worked with the in-country resident advisors to provide assistance in program design, curriculum design, teaching, evaluation, quality assurance, direct funding, and fundraising. CDC staff worked collaboratively with FELTP program staff who were either host country MOH staff or host institution personnel and were led by a host country national that was the FELTP program director. In 2007, CDC began to maintain a tracking system for all field epidemiology training programs globally with information on participant numbers and outputs that was compiled annually into a report that is disseminated to key stakeholders [22].

In 2008 we defined a series of critical outcome measures for FELTPs to better align them with their public health surveillance and response system strengthening mission [15,23]. The following critical outcomes should be increasingly evident in the host country's MOH if the FELTP is successfully contributing to its mission: a) Functional and robust public health surveillance systems, often beginning with notifiable disease surveillance and then non-communicable disease surveillance systems; b) Prompt and effective response to public health emergencies including disease outbreaks and other public health threats; c) A culture of evidence-based decision making in public health whereby programmatic decisions are based on scientifically sound data; d) A strengthened and motivated public health workforce composed of FELTP graduates of the 2-year program and the short courses conducted for frontline (i.e., sub-national, regional, provincial) public health workers; e)Evidence that the FELTP is contributing to a reduction in morbidity and mortality from priority disease conditions.

## Results

**Figure 1:**
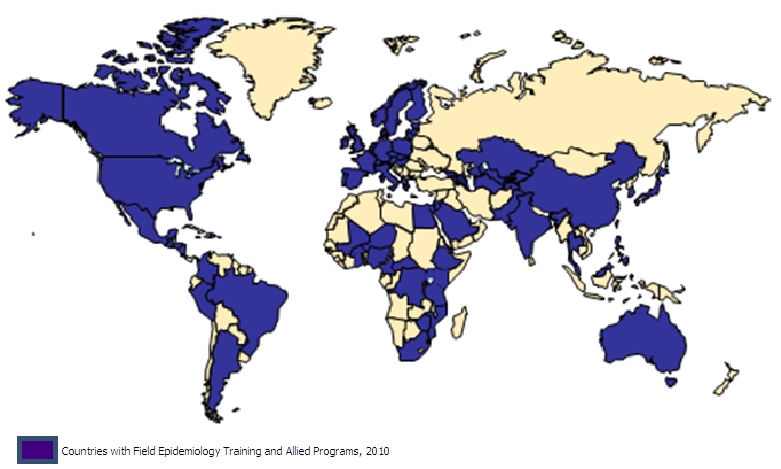
Field Epidemiology Training and allied Programs, 2010

In 2004, there were three 2-year field epidemiology training programs in sub-Saharan Africa: Kenya (which had a laboratory training component), Uganda, and Zimbabwe. By the end of 2010, there were 12 programs covering 17 countries, including Southern Sudan. The additional FELTPs include two regional programs in West Africa (with participants from Burkina Faso, Mali, Niger, and Togo, and headquartered in Ouagadougou) and Central Africa (with participants from Cameroon, Central African Republic, and Democratic Republic of the Congo, and headquartered in Yaoundé). Seven additional single-country FELTPs were created in Ethiopia, Ghana, Mozambique, Nigeria, Rwanda, South Africa, and Tanzania. The programs are in countries representing the three main official languages in the sub-Saharan region (English, French, and Portuguese). Figure 1 shows all the field epidemiology training programs in the world as of 2010.

Between 1993 and 2010 the 12 programs (10 FELTPs and two PHSWOWs) enrolled 743 public health professionals into the 2-year program, including graduates and current trainees in 2010. Of the 743, 624 (84%) were admitted into the field epidemiology training track, 95 (12.8%) were in the public health laboratory epidemiology track, 24 (3.2%) were in the veterinary epidemiology track (Additional material 1). The countries covered by the programs accounted for approximately 637 million (73.6%) of the estimated 865 million inhabitants of the sub-Saharan region in 2010. By the end of 2010 at least 1000 frontline public health workers had been trained in several types of short courses in the countries with FELTPs.

**Additional material 1:** Participants (Graduates and Current Trainees) of 2-year Public Health Schools Without Walls (PHSWOWs) and Field Epidemiology and Laboratory Training Programs (FELTPs) in Sub-Saharan Africa in 2010

PHSWOW or FELTP	Country or Countries	Year PHSWOW or FELTP Began	Country or Region Population in 2010 (millions]	Cumulative PHSWOW or FELTP Participants in 2010	Cumulative Participants per million 2010 population	Field Epidemiology Track (Degree offered)	Laboratory Epidemiology Track (Degree offered)	Veterinary Epidemiology Track (Degree offered)	Source of Startup Funding
1. Zimbabwe PHSWOW	Zimbabwe	1993	12.6	166	13.2	166(MPH)	n/a	n/a	Rockefeller Foundation
2. Uganda NISWOW	Uganda	1994	33.8	284	8.4	284 (MPH)	n/a	n/a	Rockefeller Foundation
3. Kenya FELTP	Kenya, South Sudan	2004	48.3	74	1.5	49 (MSc Applied Epidemiology)	25 (MSc Applied Epidemiology and Laboratory Management	n/a	Ellison Medical Foundation
4. South Africa FELTP	South Africa	2006	49.9	41	0.8	24 (MPH Field Epidemiology)	17 (MPH Field Epidemiology and Laboratory Management)	n/a	PEPFAR
S. Ghana FELTP	Ghana	2007	24.0	28	1.2	10 (MPhil Applied Epidemiology)	9 (MPhil Applied Epidemiology and Laboratory Management	9 {MPhil Applied Epidemiology)	USA ID
6. Tanzania FELTP	Tanzania	2008	45.0	33	0.7	20 (MSc Applied Epidemiology)	13 (MSc Applied Epidemiology and Laboratory Management	n/a	PEPFAR, USA ID, PMI
7. Nigeria FELTP	Nigeria	2008	158.3	26	0.2	12 (MSc Field Epidemiology)	6 (MSc Field Epidemiology and Laboratory	8 (MSc Veterinary Field Epidemiology)	PEPFAR, USA ID
							Management)		
8. Ethiopia FELTP	Eihiopia	2009	85.0	35	0.4	29 (MPH)	6 (MPH)	n/a	PLPhAR
9. Rwanda FELTP	Rwanda	2010	10.4	15	1.4	(MSc Applied Epidemiology)	3 (MSc Applied Epidemiology and Laboratory Management	3 (MSc Applied Epidemiology)	PLPhAR
10. West Africa FELTP	Burkina Fa so, Mali, Togo, Niger	2010	54.1	12	0.2	4 (MPH)	4 (MPH)	4 (MPH)	USAID
11. Mozambique FELTP	Mozambique	2010	23.4	11	0.5	5 (MSc Applied Epidemiology)	6 (MSc Applied Epidemiology and Laboratory Management	n/a	PEPFAR
12. Central Africa FELTP	Democratic Republic of Congo, Cameroon, Central African Republic	2010	92.6	18	0.2	12 (Master's in Field Epidemiology)	6 (Master's in Field Epidemiology and Laboratory Management	n/a	BMGF USAID
		TOTAL	637.4	743	1.2	624	95	24	

FELTP = Field Epidemiology and Laboratory Training Program, FETP= Field Epidemiology Training Program

PEPFAR = US President's Plan for Emergency AIDS Relief; PMI= US President's Malaria Initiative; USAID = US Agency for International Development; BGMF= Bill and Melinda Gates Foundation

MPH = Masters of Public Health; MSc = Masters of Science; MPhil = Masters of Philosophy

There were approximately 0.86 public health professionals who had participated in a 2-year FELTP or PHSWOW per million inhabitants in sub-Saharan Africa. In the 17 countries with programs there were approximately 1.2 FELTP or PHSWOW participants per million inhabitants, which is less than the estimated minimum coverage of 3-5 field epidemiologists per million inhabitants of a country [23]. All the sub-Saharan programs award a master's degree to participants upon completion of university requirements, some programs provide the same type of degree to field epidemiologists, public health laboratory epidemiologists, and veterinarians whereas other programs have different track-specific degrees (Table 1).

Of the 12 programs started between 1993 and 2010, six (50%) received startup funding from PEPFAR, and five (42%) received funding from USAID. Other funders included the Bill and Melinda Gates Foundation, and PMI. Host country MOHs (and ministries of agriculture for programs with veterinarians) also contributed significant resources, usually in kind (e.g., space for the program, ancillary facilities, and field sites). CDC provided supplementary funding through various initiatives for example the Global Disease Detection program in Kenya from 2006 and PEPFAR provided some support to the Zimbabwe PHSWOW [16,24]. The donor cost of implementing an FELTP ranges between US $ 1-2 million per year at inception. These costs reduced with time as expatriate staff left and the program transitioned to local staff (usually graduates of the initial cohorts) and host government funding began to replace donor funding. The costs associated with implementation include: a) resident costs which include university tuition, stipends, books, computers, and research costs; b) program costs which include emergency response, local and international travel, program office operations, field supervision visits, field site maintenance; and c) technical assistance (from Atlanta, which supports the country team); and d) resident advisor salary and support.

## Discussion

In 2004 there were three 2-year field epidemiology training programs in sub-Saharan Africa, two PHSWOWs and one FELTP. By the end of 2010 there were 12 programs, two PHSWOWs and 10 FELTPs, representing a fourfold increase in 6 years. This increase coincided with PEPFAR and USAID support of health systems strengthening and efforts to roll out WHO-AFRO's Integrated Disease Surveillance and Response strategy [4,25]. Development of a standardized process guided by FELTP host countries’ MOHs and involving all key stakeholders was critical for program implementation. Linking field epidemiology training first with the development of public health laboratory epidemiology as a new competency, and then training veterinarians to specifically address the diseases at the human-animal interface broadened the public health workforce available to operate multi-disease surveillance and response systems. Including a strategy to train frontline public health workforce through short courses also strengthened basic multi-disease surveillance and response systems with 2-year FELTP graduates often leading other trained public health professionals in joint field investigations. The 10 FELTPs and two PHWOWs had enrolled a total of 743 public health professionals in their 2-year programs by the end of 2010.

Field epidemiology training programs have been the basis on which public health surveillance and response systems have been developed around the world. CDC's EIS program, now in its 60th year has been the backbone of CDC and US State health departments’ surveillance and response systems. Similarly FELTPs are proven strategies for developing public health surveillance and response systems and can be adapted to address various public health issues. The link between 2-year training and short courses allows for flexibility to develop different but complementary skill sets within a country. Some countries, particularly those emerging from conflict situations may not be able to develop 2-year degree granting FELTPs but may be able to conduct FELTP short courses while sending a few public health professionals to a nearby FELTP to gain the competencies to start their own program. This strategy is currently underway between Southern Sudan and the Kenya FELTP, and Sierra Leone and the Ghana FELTP.

The long-term success of FELTPs depends on their sustainability. The Uganda and Zimbabwe programs have sustained since the 1990s but the path to sustainability is not pre-determined and requires several issues to be in sync. The PHSWOWs in Uganda and Zimbabwe were the first programs of their nature and were designed to develop a specific cadre of public health professionals who were virtually guaranteed of jobs utilizing their new competencies upon graduation. Both programs also included substantial training in health services management, and many graduates ended up heading district, provincial and national public health programs. FELTPs, which are developed with a closer link than PHSWOWs to development of national multi-disease surveillance and response systems and institutionalization of rapid response to public health emergencies, may require a redesign of existing public health systems to develop career paths for field epidemiologists and public health laboratory epidemiologists. Whereas the initial graduates from FELTPs can be absorbed into existing positions at national and sub-national level, the long-term sustainability of these programs requires the development of specific positions that can utilize their training appropriately. Increasingly countries are being encouraged to develop national public health institutes which can be a natural location of an FELTP at the central level [26].

Continued funding for FELTPs, which have substantial field work as part of their training strategy is an ongoing challenge. PEPFAR and USAID have provided significant support for FELTP development and starting planned programs (in Angola, Zambia, Namibia, and Botswana) is currently contingent on obtaining PEPFAR funding. Partnerships with private sector organizations and other potential funders for FELTPs will have to be developed to sustain existing programs beyond their initial funding and to start new programs where none exist. Africa in general has an improving economic climate as its natural wealth increases in value and as an African middle class develops there will likely be a need and possibly a demand for improvements in public health surveillance and response systems which can be developed and operated by locally trained FELTP graduates with funding from national governments or domestic non-governmental organizations.

Traditionally FETPs around the world typically had limited interaction with universities. However all FELTPs and PHSWOWs in sub-Saharan Africa have links with a local university and all grant master's degrees when participants complete the university requirements, which usually includes successfully defending a master's dissertation. This is a common key requirement by all the FELTP steering committees, mainly to enhance the career prospects of the FELTP graduates. Universities also provide an academic anchor for the FELTP which leverages the credibility of the universities as the program starts up. The university faculty assists in teaching the didactic components of the course and the FELTP staff strengthens the university faculty by showing them how the field-based training model works, creating shared value between the FELTP and the university. Each FELTP student has an academic supervisor or mentor from the university in addition to the program and field site supervisors. Involvement with the universities ensures that the courses are offered on time every year through a competitive selective process which helps in ensuring sustainability.

There is an ongoing discussion on how many FELTP and similarly trained public health workers are required to implement robust multi-disease surveillance and response systems in developing countries. Some authors have suggested that 3 to 5 FELTP graduates per million inhabitants in a country are needed, with a mix of field epidemiology, public health laboratory epidemiology, and veterinary epidemiology [23]. Based on this estimate sub-Saharan Africa would need between 2,600 and 4,500 FELTP graduates or similarly trained public health personnel for its population figures in 2010. Uganda with a population of 34 million in 2010 would require between 100 and 170 graduates and Zimbabwe would require a maximum of 65 graduates for a 13 million population. Both Uganda and Zimbabwe have exceeded these targets, and have a high retention of their graduates but still have challenges in their public health surveillance and response systems and continue to conduct training in their programs to strengthen public health systems [27]. We believe that beyond the number of FELTP and PHSWOW graduates there is a complementary need to develop functional and networked multi-disease surveillance and response units at national, provincial, and district levels. These units would need to be operated by a mix of FELTP and PHSWOW graduates and short course participants. It has been suggested that with functional networked multi-disease surveillance and response units, fewer graduates may be needed to operate a basic but robust public health surveillance and response system [23]. We suggest that a goal of having at least one functional multi-disease surveillance and response unit per million inhabitants in a country is worthwhile and feasible in sub-Saharan Africa. These units which would be operated by FELTP graduates and short course participants would be networked to form a multi-disease surveillance and response system for the country.

We have described how a standardized process led to the development of 10 FELTPs in sub-Saharan Africa from 2004 to 2010 but at least four challenges need to be noted. First, while we believe that all these FELTPs will be sustainable like the Uganda and Zimbabwe programs, we cannot be absolutely certain. Nevertheless, the environment that led to the creation of these programs in response to a need for a locally trained public health workforce that can operate disease surveillance and response systems for multiple conditions is likely to persist. We also believe that involvement of a high level MOH-led Steering Committee to oversee FELTP activities is likely to maintain these programs. Second, the FELTP model is still a relatively new endeavor that attempts to link public health laboratory epidemiology and field epidemiology workforce development which will need to be adapted to suit particular country environments, especially given the different career paths of the graduates of these programs. Third, the FELTPs being field-based programs require competent, committed field supervisors and field sites that are suitably equipped to host trainees with substantial funding for field operations. This can be a challenge when the program is starting as very few people in the country are familiar with this training model. If funding for field sites diminishes there may be a reduction in their quality and the overall quality of the FELTP, which depends on maintenance of appropriate field-based training. Finally, the link between FELTPs and the development and funding of the appropriate structures at national and sub-national level to absorb FELTP graduates requires ongoing work with non-health stakeholders (e.g., the legislature, the ministries of finance and public service, and the private sector).

## Conclusion

The FELTP model is one proven strategy for developing a locally trained multi-disciplinary and cohesive public health workforce that can operate several public health surveillance and response systems. We have shown this strategy can be implemented in sub-Saharan Africa, an area of the world that has numerous public health challenges–but is also experiencing an economic renaissance which will likely lead to a greater demand for strong public health surveillance and response systems to protect communities. We believe that now is the time for all sub-Saharan countries to invest in public health workforce development by implementing FELTPs or by sending public health professionals to a nearby FELTP, and to use the graduates to develop the systems and the structures that will enable a public health renaissance to accompany the economic renaissance.

## Additional material

Participants (Graduates and Current Trainees) of 2-year Public Health Schools Without Walls (PHSWOWs) and Field Epidemiology and Laboratory Training Programs (FELTPs) in Sub-Saharan Africa in 2010
